# Male fertility and semen quality are decreasing – Do we have the expertise to deal with this challenge?

**DOI:** 10.1111/aogs.14693

**Published:** 2023-11-01

**Authors:** Antti Perheentupa, Jorma Toppari

**Affiliations:** ^1^ Department of Obstetrics and Gynecology Turku University Hospital Turku Finland; ^2^ Research Center for Integrative Physiology and Pharmacology, and Center for Population Health Research, Institute on Biomedicine University of Turku Turku Finland; ^3^ Department of Pediatrics Turku University Hospital Turku Finland

Scandinavian countries have been very active in scientific research within the field of andrology, particularly high profile studies on male fertility and changes in semen quality.^1^, ^2^ It has become a well‐recognized fact that at least according to the observed changes in semen quality, male fertility has decreased significantly in recent decades.^3^

Total fertility rate (TFR; the average number of live births per woman) in Finland (1.37) is far below 2.1, which is the rate necessary to sustain a population size. The other Nordic countries are not doing significantly better (Iceland 1.72, Denmark 1.67, Sweden 1.66, Norway 1.48).^4^ It is clear that socioeconomic reasons are an important determinant for this rather alarming development, but medical reasons cannot be ignored either. More and more adults decide not to have children at all. At the same time, childbearing is left for later fertility years and this results in smaller families (fewer children) than the couples wish for. The likelihood of spontaneous pregnancies as well as those initiated following assisted reproductive techniques (ART) decreases significantly and the risk of miscarriage increases as women approach 40 years of age.^5^, ^6^ The effect of age on male fertility is far more subtle.^7^ The rising age of parents‐to‐be is clearly reflected in the number and proportion of children born following assisted reproduction, mainly by in vitro fertilization (IVF) and intracytoplasmic sperm injection (ICSI). Denmark has the highest proportion of children born following assisted reproduction in Scandinavia, one child out of 10 children; the 10% of children born following ART is very high in comparison with other countries.^8^, ^9^ The other Nordic countries have an increasing trend of ART pregnancies. Despite the relatively high proportion of all children born with ART, it does not have a significant role in the TFR, and the challenges of decreasing fertility cannot be solved by ART alone.^10^

It is important to address the fact that whereas one‐third of infertility is considered to be caused by male factors only, another third is considered to be caused by male and female factors, so male factors affect the subfertility of a couple in 50% of the cases. For more than 20 years, ICSI has been available to treat the most severe cases of male infertility. However, the development and success of ICSI resulted in quiescence in research aiming at correction of male infertility/improving male fertility. Thus, whenever even a small amount of sperm was available, that was considered sufficient to go ahead with ICSI treatment.

Andrology is the medical speciality that also deals with male fertility and infertility. In several European countries, authorities demand that each licensed infertility center has a designated certified andrologist in the staff. To fulfill this demand, official training programs for andrology exist in North America and multiple countries in Europe as well as elsewhere. Both the American Society for Reproductive Medicine (ASRM) and the European Society for Human Reproduction and Embryology (ESHRE) have a special interest group for andrology. Particularly in North America, but also in many European countries, urologists play an integral part in treating male infertility. Further training in andrology is common and well trained andrologists have little difficulty becoming employed in different units that specialize in fertility treatments. However, it seems clear from discussions with Scandinavian colleagues that the urologists in the Nordic countries are largely oriented in the surgical treatment of prostate cancer and other urological cancers. The training curriculum for urology at the Turku University Hospital does not include fertility‐related topics.

The European Academy of Andrology (EAA) was founded in 1992. EAA has 27 accredited training centers in Europe, three in Scandinavia (Copenhagen, Malmö, Stockholm). In Finland, The Finnish Medical Association has a formal curriculum for interested individuals to become experts in andrology at the end of a 2‐year training including sections on urology and gynecology. During the past 20 years, only two urologists have completed this training. Currently, the Finnish Medical Association is considering the termination of this expert program due to lack of interest. In reality, the main problem is the lack of training positions for andrology inside gynecology and urology departments, and therefore possibilities to carry out the training are very challenging, which explains the lack of trainees in this field.

The current reality in Scandinavia is that male infertility is nearly completely evaluated and treated by gynecologists with an interest or formal training in reproductive medicine. The Nordic Association for Andrology (NAFA) appears to exist only on paper or rather on Facebook. Sweden and Denmark both have an active national society for andrology, and at least part of their meeting activities have been in collaboration with each other.

The EAA andrology curriculum and exam are available for those working in the EAA Centers, and a large number of Danish and Swedish colleagues have passed the EAA examination to become recognized clinical andrologists. However, only a fairly small number of these experts are working with clinical infertility centers.

As we learn more and more about factors that affect male fertility, it is becoming clear that it is important to optimize the male fertility even in cases where ART is ultimately needed. In some cases, invasive treatments may be avoided altogether. Best quality sperm is important not only to ensure pregnancy outcomes with the ART but also to make sure that our treatments result in children that are as healthy as possible. We have numerous examples of fruitful Nordic research collaborations (eg CONARTAS, ReproUnion, Turku–Copenhagen cohort studies). The results of the large registry studies of ART results and offspring have led to changes in clinical practice with little delay. How do we ensure that the male issue is not only considered important but that we also train adequate numbers of andrologists to put the available knowledge into clinical practice? We should support investment in better training of andrologists in all Nordic countries to improve our fertility treatments and the entire healthcare available.
